# Transcatheter Edge-to-Edge Repair of a Degenerated Mitral Valve in Complex Congenital Heart Disease

**DOI:** 10.1016/j.jscai.2025.102618

**Published:** 2025-03-07

**Authors:** Ka-Chun Un, Cheung-Chi Lam, Hay-Son Chen, Chun-Ka Wong, Ho-On Alston Conrad Chiu, Yui-Ming Lam, Gilbert H.L. Tang, Kwong-Yue Eric Chan

**Affiliations:** a Cardiology Division, Department of Medicine, Li Ka Shing Faculty of Medicine, The University of Hong Kong, Hong Kong SAR; b Cardiology Division, Department of Medicine, Queen Mary Hospital, Hong Kong SAR; c Cardiology Unit, Department of Paediatrics and Adolescent Medicine, Hong Kong Children’s Hospital, Hong Kong SAR; d Department of Cardiovascular Surgery, Mount Sinai Health System, New York, New York; e Cardiac Medical Unit, Grantham Hospital, Hong Kong SAR

**Keywords:** Adult congenital heart diseases, complex cyanotic heart diseases, transcatheter mitral valve repair

Patients with complex congenital heart diseases are often considered to be high risk for open heart surgery; however, atrioventricular valve pathologies are not uncommon, especially in those with single ventricular physiology. Despite valve replacement being widely reported, there are only a few reported cases regarding transcatheter valvuloplasty, as the experience is still limited worldwide.^1^^,^^2^

## Case description

**Figure 1:**
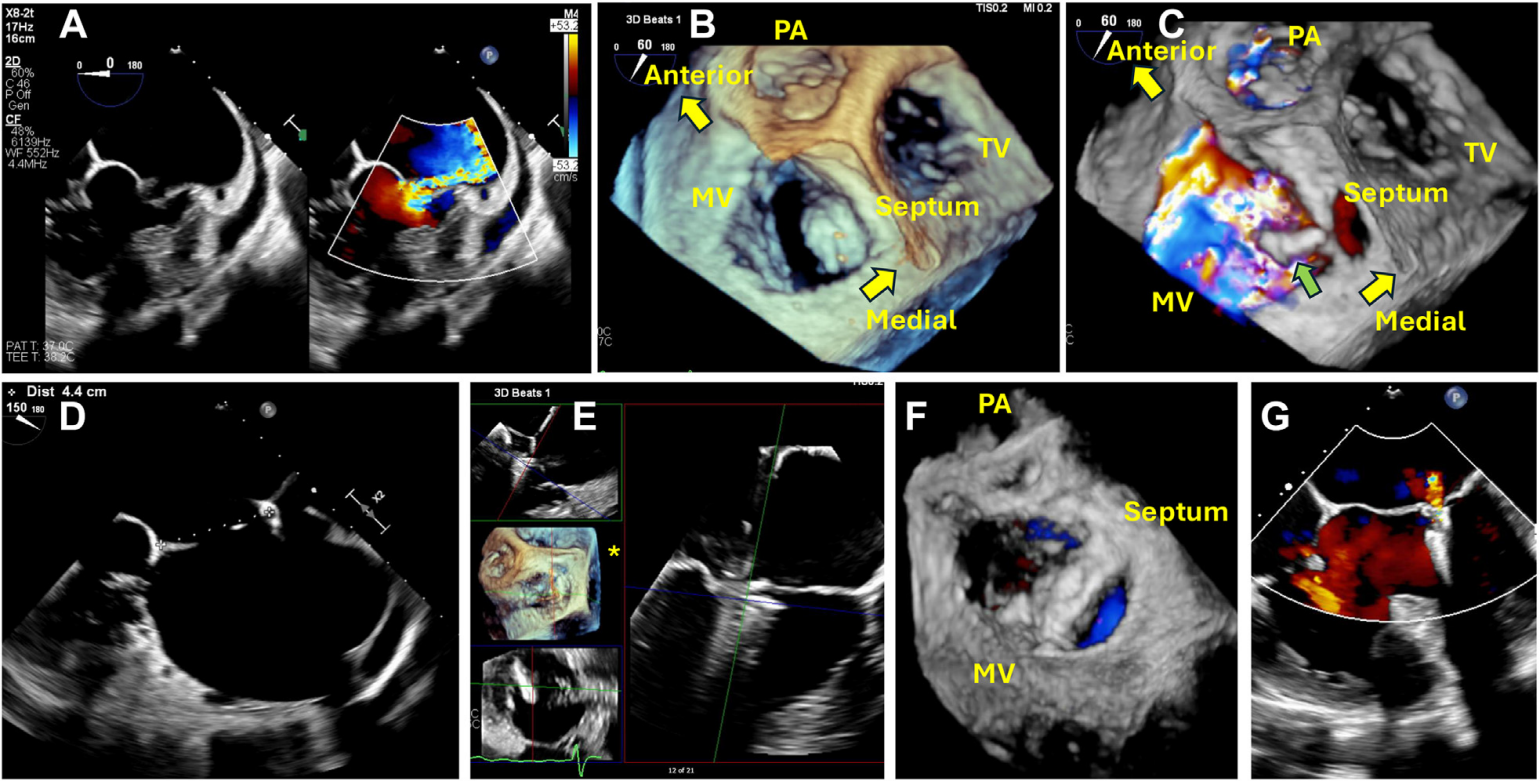
**Transcatheter edge-to-edge repair of a rotated mitral valve in complex congenital heart disease.** (A-C) Abnormal clockwise rotation of the mitral valve and severe mitral regurgitation (MR) due to flail posteroseptal segments (green arrow). (D) Multiple atrial septal defects (ASDs), one of the ASDs was crossed with height of 4.4 cm from mitral annular plane. (E) Intra-operative multi-planar reconstruction with counterclockwise 90° rotation (asterisk). (F) Satisfactory tissue bridge post-MitraClip deployment. (G) Reduction of MR post-procedurally. MV, mitral valve; PA, pulmonary artery; TV, tricuspid valve.

A 55-year-old woman with atrial fibrillation, unrepaired double-outlet right ventricle, transposition of great arteries, severe pulmonary stenosis, large unrestrictive ventricular septal defect extending from inlet to outlet, and multiple atrial septal defects (ASDs) presented with acute heart failure. Echocardiography showed a left ventricular ejection fraction of 60% and clockwise 90° rotated mitral valve in septal and lateral bileaflets configuration, with a flail segment over posteroseptal region, resulting in severe eccentric mitral regurgitation (Figure 1A-C). The patient had known congenital heart diseases since childhood but has defaulted follow-up for more than 30 years. The heart team considered the patient technically difficult for biventricular (Rastelli type) repair due to straddling mitral valve and recommended transcatheter edge-to-edge repair (TEER) as palliation.

The MitraClip G4 Steerable Guide Catheter (Abbott Structural Heart) was inserted from the right femoral vein through one of the ASDs, achieving a height of 4.4 cm from the mitral annular plane (Figure 1D). The baseline mean left atrial pressure was 15 mm Hg with giant V waves at 31 mm Hg. The echocardiographic images were rotated counterclockwise 90° throughout the procedure to resemble “normal” anatomy (Figure 1E). The first MitraClip XTW was placed at the usual “A3/P3” segments. Clockwise rotation of the guide was used to reach the medial commissure, and the M knob was turned with the guide withdrawn to reach the anterior leaflet. Another MitraClip XTW was further positioned anterior to the previous MitraClip (Figure 1F). Mitral regurgitation reduced to mild (Figure 1G). Mean left atrial pressure improved to 4 mm Hg with loss of giant V waves at 6 mm Hg. Transmitral mean gradient remained 2 mm Hg before and after the procedure. Apixaban was resumed postprocedurally for her atrial fibrillation.

This case demonstrates the potential application of TEER of malformed atrioventricular valves in a patient with complex congenital heart disease. An unusual anatomy of the valves may be encountered, and interventionists and imagers should anticipate the challenges and be ready to handle them.

## Supplementary material

Video1 Abnormal clockwise rotation of the mitral valve.

Video2 Reduction in mitral regurgitation post-procedure.
